# Acute Effects of Caffeine Intake on Psychological Responses and High-Intensity Exercise Performance

**DOI:** 10.3390/ijerph18020584

**Published:** 2021-01-12

**Authors:** Raúl Domínguez, Pablo Veiga-Herreros, Antonio Jesús Sánchez-Oliver, Juan José Montoya, Juan José Ramos-Álvarez, Francisco Miguel-Tobal, Ángel Lago-Rodríguez, Pablo Jodra

**Affiliations:** 1 Centro Adscrito a la Universidad de Sevilla, Escuela Universitaria de Osuna, 41640 Osuna, Spain; rauldh@euosuna.org or or; 2 Departamento de Educación Física y Deporte, Universidad de Sevilla, 41013 Sevilla, Spain; 3 Studies Research Group in Neuromuscular Responses (GEPREN), University of Lavras, 37200-000 Lavras, Brazil; 4 Faculty of Health Sciences, Universidad Alfonso X El Sabio, 28691 Madrid, Spain; pveigher@uax.es; 5 Departmento de Motricidad Humana y Rendimiento Deportivo, Universidad de Sevilla, 41013 Sevilla, Spain; sanchezoliver@us.es; 6 Faculty of Medicine, School of Medicine of Physical Education and Sport, Madrid Complutense University, 28040 Madrid, Spain; jjmontoya@ucm.es (J.J.M.); jjramosa@ucm.es (J.J.R.-Á.); miguelto@ucm.es (F.M.-T.); 7 Faculty of Health Sciences, Universidad Isabel I, 09004 Burgos, Spain; 8 Faculty of Education Sciences, University of Alcalá, 19001 Guadalajara, Spain; pablo.jodraj@uah.es

**Keywords:** ergogenic aid, sport supplement, sport performance, Wingate test, rate of perceived exertion, subjective vitality, mood

## Abstract

Objective: The aim of this study was to investigate the effects of caffeine supplementation on: (i) psychological responses of subjective vitality and mood; (ii) performance through a Wingate test; and (iii) rate of perceived exertion (RPE) reported after a Wingate test. Methods: Fifteen male participants (22.60 ± 2.16 years) ingested 6 mg·kg-1 of caffeine or placebo (sucrose) supplementation in two experimental sessions. After 60 min from supplement intake, participants fulfilled two questionnaires, which measured subjective vitality and mood state, respectively. Subsequently, participants’ performance was assessed through a Wingate test, which was followed by measurements of RPE at general, muscular, or cardiovascular level. Results: Caffeine supplementation increased some components of mood, as assessed by profile of mood states (POMS) (tension and vigor dimensions) and subjective vitality profiles, which were followed by a greater maximum power, average power, and lower time needed to reach maximum power during the Wingate test. Moreover, lower RPE, both at muscular and general levels were reported by participants after the Wingate test. Conclusions: These results suggest that caffeine supplementation exerts positive effects both in psychological and physical domains in trained subjects.

## 1. Introduction

A performance improvement of the order of 1.6% makes the difference between the gold medalist and the athlete finishing at fourth place at the Olympic Games [1]. This performance feature at maximum competitive level might explain the high prevalence of sport supplements consumption among athletes. Among currently available sports supplements, caffeine consumption has increased considerably since 2004, when it was removed from the list of banned substances for sports [2] based on its ergogenic effect [3]. Initially, in the 1970s, the ergogenic effect of caffeine was attributed to enhanced lipolysis and enhanced muscle glycogen spare [4]. Currently, caffeine’s ergogenic effect is explained by its antagonistic role on adenosine receptors [5]. Being similar in structure to adenosine, caffeine has the capacity of blocking adenosine receptors A_1_, A_2a_, and A_2b_ [6]. Since there are numerous adenosine receptors at the neuromuscular level [7], caffeine increases neuromuscular recruitment [8], whereas at the muscular level, caffeine induces calcium release from the sarcoplasmic reticulum and can also inhibit its reuptake [9], resulting in translocation of glycogen phosphorylase b into isoform a, via an increased calcium bioavailability [10]. Furthermore, caffeine also increases the activity of the enzyme phosphofructokinase, thereby maximizing glycolysis activity [11]. 

Interestingly, athletic performance may be enhanced not only through peripheral mechanisms, but also via central mechanisms [12]. At a central level, thanks to its antagonistic role on adenosine receptors [5], caffeine counteracts the inhibitory function of adenosine on the release of several excitatory neurotransmitters in the brain [13], particularly dopamine [14], thus alleviating the effect of adenosine on the excitatory activity of the central nervous system [5]. Therefore, caffeine may alleviate the effects of adenosine on increased feelings of lack of energy, increased rate of perceived exertion (RPE), and subjective tiredness and fatigue [15]. Since caffeine also increases the release of some excitatory neurotransmitters, such as dopamine and noradrenaline [16], a potential central ergogenic effect for caffeine supplementation has been considered [12], with caffeine supplementation having a positive incidence on mood [17,18], increasing alertness level and reducing the feeling of fatigue [19,20]. 

Cognitive function plays an important role in athletic performance [21]. It has been observed that caffeine supplementation leads to a more favorable mood profile [17,18], which positively influences exercise performance [22] and reduces RPE during exercise [23,24]. However, most of the studies performed on caffeine supplementation have mainly focused on its ergogenic effects on physical performance in exercises that predominantly rely on the oxidative metabolism, such as endurance sport modalities [25,26], as well as sport modalities where performance relies on a mixed metabolism, such as racquet sport [27], combat sports [28], or team sports [29]. Nevertheless, there is growing interest in the effects of caffeine supplementation on exercises predominantly relying on the glycolytic metabolism. In this regard, the Wingate test is one of the most valid and reliable tools used for measuring anaerobic performance [30], since it allows to evaluate muscle capacity to generate power through anaerobic energy systems [31]. Interestingly, an ergogenic effect of caffeine supplementation on a Wingate test has been recently reported [32]. However, only few studies have directly analyzed the effects of caffeine supplementation at the psychological level. Considering a more integrative approach, the aim of this study was to examine the acute effects of caffeine intake on: (i) psychological responses of subjective vitality and mood; (ii) physical performance during a high-intensity, highly glycolytic exercise test (Wingate); and (iii) RPE reported after the Wingate test.

## 2. Materials and Methods

### 2.1. Participants

A total of 15 male volunteers (age: 22.60 ± 2.16 years; height: 1.77 ± 0.04 m; mass: 78.11 ± 10.63 kg; body mass index: 24.99 ± 2.61 kg/m^2^) participated in this study. All participants were undergraduate students in the Physical Activity and Sport Sciences program and had resistance training experience. Participation was voluntary, with the following inclusion criteria established in an information session: (a) having completed at least three sessions per week of strength training within the past 18 months; (b) a bench press one-repetition maximum (1 RM) greater than body weight, and full squat 1 RM 1.5 times body weight; (c) no nutritional supplements taken in the three months before the study onset; (d) no smoking; (e) no cardiovascular, respiratory, metabolic, neurological, or orthopedic disorders that could affect cycle ergometer performance; (f) not considered an elite athlete; and (g) experience with the Wingate test, having performed at least one test within 3 months before study onset. During the information session, participants were informed about the aim and procedures of the study, including the diet to be followed and caffeine intake, and then signed written consent to participate in the study. The study protocol was approved by a local Ethics Committee. 

### 2.2. Study Design

The study design was randomized, double-blind, placebo-controlled, cross-over design. Subjects were randomized in a 1:1 ratio using a computer-generated randomization (Research Randomizer, www.randomizer.org) either to a group starting session 1 with caffeine supplementation or with placebo. Experimental sessions were performed in the Exercise Physiology laboratory 72 h apart. The sessions were scheduled at the same time of day (±0.5 h) to avoid possible circadian rhythm effects. Subjects were instructed to refrain from any type of physical exercise for 72 h before the first session and until the end of the study.

Upon arrival at the laboratory for each session, participants were given a caffeine or placebo supplement. Sixty minutes after the intake of the supplement, they completed the questionnaires profile of mood states (POMS) and subjective vitality scale (SVS). Following the questionnaires, and a brief warm-up, participants performed a Wingate test on a cycle ergometer (Ergomedic 828E, Monark Exercise AB, Vansbro, Sweden). Immediately after the test, they graded their exertion using a standardized rate of perceived exertion (RPE) scale [33]. 

### 2.3. Supplementation Protocol and Diet Control

Subjects arrived at the laboratory 75 min before initiating the Wingate test. As soon as they arrived, they were provided with either the caffeine supplement (6 mg·kg^−1^) or placebo (6 mg·kg^−1^ of sucrose). Caffeine supplements were provided in #1 non-transparent red capsules (Guinama S.L.U, 0044634, La Pobla de Valbona, Spain). Capsules were prepared following the standard work procedure using a semiautomatic manual filling machine Capsunorm 2000 (Tecny-Farma SLU, Miranda de Ebro, Spain). The timing of supplement intake was based on the fact that peak blood caffeine levels are reached one hour post-ingestion [34] and on the results of a disaggregation quality assay of 13.4 min.

As nutritional intake affects energy, participants were provided with detailed dietary instructions to follow from the beginning of session 1 to the completion of session 2. These instructions were important to ensure that participants had a similar macronutrients diet composition (60% carbohydrates, 30% lipids and 10% proteins) during the study. Caffeine intake was also restricted 24 h before the study onset and the subjects were provided with a list of foodstuffs rich in caffeine (coffee, tea, mate, energizing drinks, cola drinks, chocolate drinks, and chocolate) they should avoid. Participants were instructed to record their food intake for 48 h prior to the first supplementation trial and to reproduce it prior to the second supplementation trial.

### 2.4. Profile of Mood States (POMS)

To assess participants’ mood, the profile of mood states (POMS) questionnaire was used in its reduced version [35], translated into Spanish and validated by Fuentes et al. (1995) [36]. Participants graded a set of 29 adjectives items related to mood on a Likert scale from 0 (not at all) to 4 (extremely) in reply to the question “How do you feel at this moment?” to assess six scales: tension, depression, anger, vigor, fatigue, and confusion. 

### 2.5. Subjective Vitality Scale (SVS)

Participants’ vitality was assessed using the Spanish version [37] of the subjective vitality scale (SVS) [38]. Subjects were required to indicate their agreement with seven statements related to subjective feelings of energy and vitality using a 7-point Likert scale, where 1 meant “total disagreement” and 7 meant “total agreement”.

### 2.6. Wingate Test

A Monark cycle ergometer (Ergomedic 828E, Monark Exercise AB, Vansbro, Sweden) was used for the Wingate test. Subjects performed a standardized warm-up [39,40], consisting of 5 min of initial light cycling with the workload and cadence set by the subject, which was followed by 1 min of rest, and then 3 min of pedaling at a rate of 60 revolutions per minute, with a workload of 2 N·m·kg^−1^ and a sprint at maximum intensity in the last 5-s of each minute. After 2 min of rest, the Wingate test was started.

The Wingate test consisted of 30 s of cycling at maximum effort with a load (N·m·kg^−1^) corresponding to 7.5% of the subject’s body weight. Participants were instructed: (i) to reach the maximum revolutions per minute in the shortest time possible; and (ii) try to maintain this pedaling speed until the end of the test. 

Power was recorded during each second of the test. The following variables were subsequently calculated: the highest power value recorded during the test or peak power, the time in seconds taken to reach maximum power, mean power for the test duration, and minimum power measured as the lowest value recorded during the last 10 s of the test. 

### 2.7. Rating of Perceived Exertion (RPE)

In line with previous research [41,42], a 6 to 20 RPE scale of Borg (1978) [33] was presented as soon as participants finished the Wingate test. Accordingly, participants were first asked to report RPE regarding perceived exertion at legs (RPE_muscular_); second, participants were asked to report RPE only at cardiorespiratory level (RPE_cardio_); and finally, participants had to declare global RPE (RPE_general_), which included features from both muscular and cardiorespiratory levels. 

### 2.8. Statistical Analysis

All data are provided as mean (M) ± standard deviation (SD). The normality of the data collected was first established using the Shapiro–Wilk test. A Student’s t-test for related samples was used to compare values from the two experimental conditions (caffeine vs. placebo) for variables showing a normal distribution. For not-normally distributed data, the Wilcoxon test for paired data was used. Additionally, effect sizes (ES) were calculated through Cohen’s d as: large (d > 0.8), moderate (d = 0.8 to 0.5), small (d = 0.5 to 0.2), and trivial (d < 0.2). Significance was set at *p* < 0.05. All statistical tests were performed using the software package SPSS version 19.0 (IBM SPSS Statistics, Chicago, Illinois, USA).

## 3. Results

**Figure 1:**
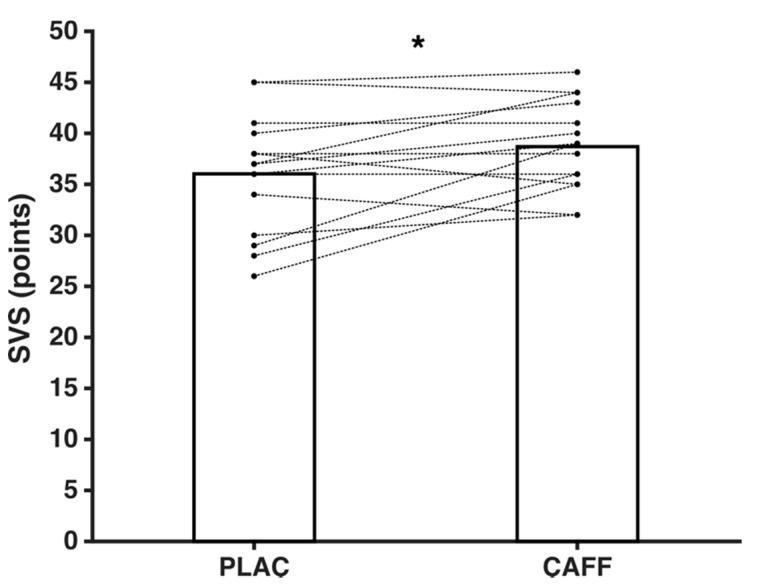
Mean subjective vitality scale (SVS) scores (thick line) and scores recorded in each participant (dotted lines). PLAC: placebo group; CAFF: caffeine supplementation group. * Significant difference between caffeine and placebo (*p* < 0.05).

After caffeine supplementation, higher values were observed for the subjective vitality scale scores (+7.41%, 38.67 ± 4.37 vs. 36.00 ± 5.77, *t*_14_ = −2.53, *p* = 0.024, ES = 0.54) (see Figure 1), and for the dimensions tension (+88.89%, 6.80 ± 5.78 vs. 3.60 ± 2.90, *z* = −2.911, *p* = 0.004, ES = 0.72) and vigor (+7.73%, 13.00 ± 3.07 vs. 12.07 ± 3.28, *z* = −1.412, *p* = 0.15, ES = 0.3) from the POMS questionnaire (Table 1). 

**Figure 2:**
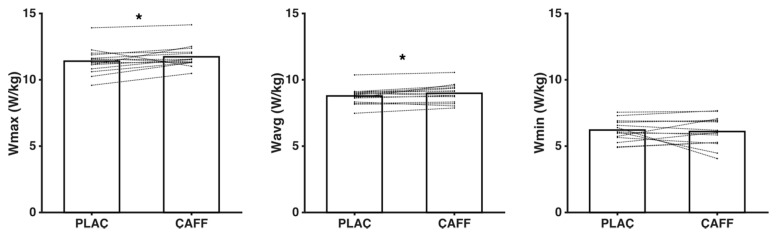
Power (W/kg) variables Wmax, Wavg, and Wmin recorded under placebo and caffeine experimental conditions. PLAC: placebo group; CAFF: caffeine supplementation group. * Significant difference for placebo vs. caffeine (*p* < 0.05).

In the Wingate test, an improvement of +2.87% for W_max_ (11.72 ± 0.85 W/kg vs. 11.39 ± 0.98 W/kg, *z* = −2.062, *p* = 0.039, ES = 0.37) and of +2.29% for W_avg_ (8.97 ± 0.70 W/kg vs. 8.77 ± 0.63 W/kg, *t*_14_ = −2.45, *p* = 0.028, ES = 0.31) was observed, without changes in W_min_ (−1.8%, 6.09 ± 1.09 W/kg, vs. 6.20 ± 0.81 W/kg, *t*_14_ = 0.49, *p* = 0.632, ES = 0.12) after caffeine supplementation (see Figure 2). In addition, it was noted that under the caffeine condition, subjects showed a significantly lower T_W_max_ (−19.08%, 7.07 ± 1.33 s vs. 8.73 ± 1.49 s, *z* = −3.108, *p* = 0.002, ES = 1.22).

**Figure 3:**
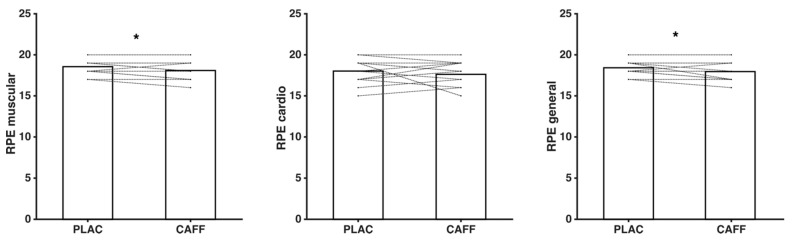
Rate of perceived exertion (RPE) (points) values observed at RPE_muscular_, RPE_cardio_, and RPE_general_ recorded under placebo and caffeine experimental conditions. PLAC: placebo group; CAFF: caffeine supplementation group. * Significant difference for placebo vs. caffeine (*p* < 0.05).

Regarding RPE, lower values for RPE_muscular_ (−2.52%, 18.07 ± 1.16 vs. 18.53 ± 0.92, *t*_14_ = 2.824, *p* = 0.014, ES = 0.45) and RPE_general_ (−2.54%, 17.93 ± 1.22 vs. 18.40 ± 0.91, *z* = −2.11, *p* = 0.035, ES = 0.45) were registered after caffeine supplementation, compared to placebo, whereas no differences were observed for RPE_cardio_ (−2.22%, 17.6 ± 1.55, vs. 18.0 ± 1.46, *t*_14_ = 0.878, *p* = 0.395, ES = 0.27) (see Figure 3).

## 4. Discussion

This study investigated the effects of caffeine supplementation on psychological (subjective vitality, mood, and RPE) and performance outcomes during a highly glycolytic task (Wingate test). The main findings of this research provide evidence of an ergogenic effect of caffeine supplementation on physical performance (improving W_max_, W_avg_, and T_W_max_), subjective vitality, mood profile (tension and vigor), and RPE (RPE_muscular_ and RPE_general_).

Greater tension values reported in the present study after caffeine supplementation are in line with previous observations in judokas [43,44,45], female team-game players [20], and elite and well-trained recreational athletes [41]. Although it has been previously suggested that greater tension values observed after a large caffeine dose could lead to anxiety-mediated negative effects on performance [46], it may also reflect an optimization of an athlete’s state of preparation to tackle a physical test, since the association between tension and performance follows an inverted U-shaped function, whereby extreme low and high tension levels are detrimental to elicit the individual’s optimal performance zone [47]. In this regard, higher predisposition to approaching a task has been previously observed in subjects reporting greater tension values after caffeine supplementation [20,48,49,50]. Subjective vitality is associated with feelings of personal energy, and the enthusiasm towards an activity [38], and it is a consequence of autonomously-motivated behavior [51]. Results from the present study show greater SVS after caffeine supplementation, which is in agreement with observations from Da Silva et al. (2015) [52], who reported that caffeine supplementation increased an athlete’s readiness to invest in mental effort. Altogether, results found for SVS and tension suggest that caffeine supplementation leads to an optimal emotional state to approach a physical task, which may be mediated via augmented tension [42]. Furthermore, this favorable mood state was followed by a performance enhancement at the beginning of the Wingate test, increasing W_max_ (+11.7%) and T_W_max_ (−19.1%). In this regard, an effect of caffeine supplementation on mood (i.e., higher tension) followed by an enhanced W_max_ and T_W_max_ in a Wingate test has been previously reported [41,42]. Improvements observed for W_max_ and T_W_max_ could be mediated by peripheral effects, such as calcium potentiation in the myoplasm [9], which can influence myosin–actin interaction [42], and an enhanced motor unit recruitment and synchronization [53,54]; although the improved mood profile observed in this study might also explain higher power values observed at the beginning of the Wingate test.

Along with improvements on W_max_ and T_W_max_, higher W_avg_ (+2.3%) was also observed in the present study after acute caffeine supplementation, which is in line with previous reports [32,41,54]. Given that the Wingate test leads to high glycolytic demands, thus leading to great fatigue [32,55,56], the increased W_avg_ after caffeine supplementation may be mediated by an indirect enhancement of the glycolytic metabolism via an increased activity of the enzyme phosphofructokinase or a stimulation of catecholamine production [11]. However, these results may be also partially mediated by the greater vigor level (+7.8%) observed in the present study after caffeine supplementation, which is in line with previous studies reporting improved vigor and time to exhaustion at an intensity of 80%VO_2max_ [57], or the maximal number of repetitions with a load of 60% 1RM [58]. 

Greater performance for the Wingate test was accompanied by lower RPE_muscular_ and RPE_general_. These results are in line with previous research on the effects of caffeine supplementation, which reported a reduction in the relative RPE/velocity in a gradual progressive test [59], and lower RPE after a training session of 30 [60] and 90 min [61] at an intensity corresponding to 70% VO_2max_. Thus, caffeine seems to modify the relationship between workload and RPE, which could contribute to the W_avg_ improvement observed in the present study. Attending to the central governor theory [62], physiological responses are monitored via afferent feedback and regulated through subsequent efferent input to appropriate control systems. At a central level, an accumulation of adenosine in the cortex could lead to fatigue [63,64] as a result of a diminished activity of the central nervous system [5], which would in turn increase RPE. Thus, blocking the adenosine receptors in the central nervous system via caffeine supplementation may contribute to the observed ergogenic effect [65], counteracting the negative effect of adenosine on central fatigue. Additionally, caffeine improves the tissue oxygen extraction and reduces the cerebral metabolic rate of oxygen consumption [66]. Since reduced blood flow to the brain during exercise has been identified as a factor promoting fatigue [67], the effect of caffeine supplementation on RPE_general_ observed in the present study could be partially mediated by caffeine’s impact on cerebral oxygenation. Besides, the decrease of RPE_muscular_ after caffeine supplementation is in line with previous observations on participants cycling to fatigue [68]. Since caffeine’s antinociceptive properties justify its inclusion in several analgesic preparations [69], caffeine’s analgesic effect has been suggested to reduce pain during exercise, thus leading to reduced RPE_muscular_ [60]. Therefore, the effect of caffeine supplementation on RPE_muscular_ observed in the present study could be partially mediated by caffeine’s analgesic effect.

The present study has some limitations. First, although the Wingate test is a valid and reliable tool for measuring anaerobic performance [30], which allows to evaluate muscle capacity to generate power through anaerobic energy systems [31], blood lactate concentration should have been assessed as an indicator of glycolytic activity [70,71]. Second, since performance of habitual caffeine consumers may be affected when deprived from caffeine [72], participants’ habitual caffeine consumption should have been registered, and accounted for in the statistical analysis. Furthermore, caffeine consumption is different according to the sports modality practiced [73,74]; this could have conditioned the previous use of this supplement in the different subjects of the sample and, therefore their response to it. Finally, a baseline measure for mood profile and subjective perception of vitality was not performed at each experimental session, and so it should be noted when interpreting the results of the present study.

## 5. Conclusions

Our findings indicate that acute caffeine supplementation in trained subjects has positive effects on several psychophysiological mood variables and psychological responses, and on performance. Caffeine supplementation increases tension, vigor, and subjective perception of vitality, while it reduces RPE_general_ and RPE_muscular_. These responses favor an optimal psychological state that was accompanied by the ergogenic effect observed for performance variables such as W_max_, W_avg_, and T_W_max_.

## Figures and Tables

**Table 1:** Scores obtained for the different profile of mood states (POMS) scales for the two experimental conditions (caffeine, placebo)**.**

Scheme	Caffeine	Placebo	Mean Difference (%)	*p*	*ES*
Tension *	6.80 ± 5.78	3.60 ± 2.90	+88.9	0.004	0.72
Depression	1.20 ± 1.52	1.47 ± 3.09	−18.4	0.565	0.11
Anger	2.27 ± 2.81	1.27 ± 2.31	+78.7	0.088	0.40
Vigor *	13.20 ± 3.23	11.87 ± 3.04	+11.2	0.010	0.44
Fatigue	2.93 ± 2.04	3.53 ± 3.44	−17.0	0.476	0.19
Confusion	12.93 ± 4.08	13.27 ± 2.69	−2.6	0.690	0.10

Data are provided as the mean ± standard deviation. * Significant difference between caffeine and placebo (*p* < 0.05).

## Data Availability

The data presented in this study are available on request from the corresponding author. The data are not publicly available due to restrictions privacy.
